# Capacitive Organic Anode Based on Fluorinated‐Contorted Hexabenzocoronene: Applicable to Lithium‐Ion and Sodium‐Ion Storage Cells

**DOI:** 10.1002/advs.201801365

**Published:** 2018-11-02

**Authors:** Jaehyun Park, Cheol Woo Lee, Ju Hyun Park, Se Hun Joo, Sang Kyu Kwak, Seokhoon Ahn, Seok Ju Kang

**Affiliations:** ^1^ Department of Energy Engineering School of Energy and Chemical Engineering Ulsan National Institute of Science and Technology (UNIST) Ulsan 44919 Republic of Korea; ^2^ Institute of Advanced Composite Materials Korea Institute of Science and Technology (KIST) Jeonbuk 55324 Republic of Korea; ^3^ Applied Materials Institute for BIN Convergence Department of BIN Convergence Technology and Department of Polymer‐Nano Science and Technology Chonbuk National University Jeonbuk 54896 Republic of Korea

**Keywords:** contorted hexabenzocoronene, electrochemical capacitors, fluorination, high current rates, pseudocapacitors

## Abstract

Conducting polymer‐based organic electrochemical capacitor materials have attracted attention because of their highly conductive nature and highly reversible redox reactions on the surface of electrodes. However, owing to their poor stabilities in aprotic electrolytes, alternative organic electrochemical capacitive electrodes are being actively sought. Here, fluorine atoms are introduced into contorted hexabenzocoronene (cHBC) to achieve the first small‐molecule‐based organic capacitive energy‐storage cells that operate at high current rates with satisfactory specific capacities of ≈160 mA h g^−1^ and superior cycle capabilities (>400) without changing significantly. This high capacitive behavior in the P2_1_/c crystal phase of fluorinated cHBC (F—cHBC) is caused mainly by the fluorine atoms at the end of each peripheral aromatic ring. Combined Monte Carlo simulations and density functional theory (DFT) calculations show that the most electronegative fluorine atoms accelerate ion diffusion on the surface to promote fast Li^+^ ion uptake and release by an applied current. Moreover, F—cHBC has potential applications as the capacitive anode in Na‐ion storage cells. The fast dynamics of its capacitive behavior allow it to deliver a specific capacity of 65 mA h g^−1^ at a high current of 4000 mA g^−1^.

## 1 Introduction

With the demand for high‐performance electrochemical energy storage, storage cells with high capacities and fast rate capabilities are being studied for next‐generation energy‐storage systems.^1^, ^2^, ^3^, ^4^, ^5^ Although advanced rechargeable batteries have many advantages and are widely used in power applications,^3^, ^4^, ^5^ the classic intercalation mechanism hinders the attainment of high specific capacities during high rate discharge–charge processes, which has led researchers to pursue storage cells with new Li‐insertion mechanisms.^6^, ^7^ Extreme rate performance has been achieved with supercapacitor‐type cells, where the unique electrostatic double‐layer capacitor (EDLC) mechanism can fundamentally solve the rate problems and exhibit millisecond discharge–charge times.^2^, ^8^, ^9^, ^10^, ^11^ Although the positive influence of Helmholtz layers on the electrodes surface can decrease the Li‐ion adsorption time, the inherent limited capacities of supercapacitors negate the advantage of their ultrafast discharge–charge times, although much effort have been devoted to alleviating this issue.^9^, ^10^, ^11^

To satisfy the demand for high energy‐storage capacities and discharge–charge rates, pseudocapacitors have attracted interest in the field of electrochemical energy‐storage cells.^12^, ^13^, ^14^, ^15^ Fast redox reactions near the electrode surfaces without bulk phase transformations allow cells to exhibit higher capacitances and rate capabilities than conventional supercapacitors and batteries.^12^ After the first observation of pseudocapacitance in RuO_2_ anodes,^15^ various pseudocapacitive transition metal oxides (e.g., MnO_2_, Fe_2_O_3_, α‐MoO_3_, and Nb_2_O_5_) and nanostructured layered metal hydroxides (e.g., Ni(OH)_2_ and Co(OH)_2_) have been proposed and meet the requirements for higher specific capacitances.^16^, ^17^, ^18^, ^19^, ^20^, ^21^ However, several major obstacles, such as low conductivities, toxicities of materials, and high weights of metal oxide materials impede their use in pseudocapacitive electrodes.^13^, ^22^, ^23^

As a potential alternative, conjugated polymers are intriguing, as they possess high electrical conductivities and form lightweight electrodes.^24^, ^25^, ^26^ Recently, various organic materials, such as polydopamine‐derived electrodes, thiophene‐rich conjugated microporous polymers, and conjugated ladder‐structured oligomers, have been proposed and revealed their merits in Li‐ and Na‐ion cells.^27^, ^28^, ^29^ In particular, the tunable bandgap between highest occupied molecular orbital (HOMO) and lowest unoccupied molecular orbital (LUMO) accelerates the charge transfer kinetics.^24^ Nevertheless, poor cycle ability due to swelling in aprotic solvents is a major limiting factor, requiring an additional process to stabilize the conducting polymers by using recently developed methods, including the deposition of a thin carbonaceous shell^25^, ^26^ of a 3D hierarchical nanostructure of conductive polymer hydrogels (CPHs)^30^ or of a Nafion coating.^31^ As for the more stable conducting organic materials, our group recently demonstrated that contorted hexabenzocoronene (cHBC) small molecules have potential uses as anode materials for Li‐ion batteries (LIBs).^32^ A doubly concave conformation of cHBC can increase the *d*‐spacing and consequently enhance the discharge and charge rates and achieve superior cycling capabilities in LIBs without significant alterations due to aprotic electrolytes. Furthermore, cHBC shows great potential for achieving control of the HOMO and LUMO levels by doping the heteroatoms in the cHBC.^33^ However, capacitive electrodes based on small molecules have been rarely reported for electrochemical energy‐storage cells.

Herein, we report the strategic design and synthesis of fluorinated cHBC (F—cHBC) molecules for possible use as high‐capacity and high‐rate electrochemical capacitor electrodes. We have chosen the most electronegative fluorine atom to coordinate the exterior aromatic rings to control the HOMO and LUMO energy levels of cHBC. The decreased HOMO–LUMO energy levels of F—cHBC showed a highly crystalline P2_1_/c phase, which permits the development of pseudocapacitor characteristics in half‐cell architectures, providing an excellent rate capability of 7000 mA g^−1^ with a reversible capacity of ≈100 mA h g^−1^. In particular, simulation results indicated that the negative charge of the fluorine atoms in the F—cHBC crystal promotes Li accessibility, which ultimately increases the rate performance during the discharge–charge processes. Furthermore, F—cHBC exhibited superior Na storage performance and long‐term stability; these traits suggest that F—cHBC is a potential electrochemical organic electrode for Li^+^‐ and Na^+^‐ion storage cells.

## 2 Results and Discussion

### 2.1 Crystal Structure and Phase of F—cHBC Anode

**Figure 1:**
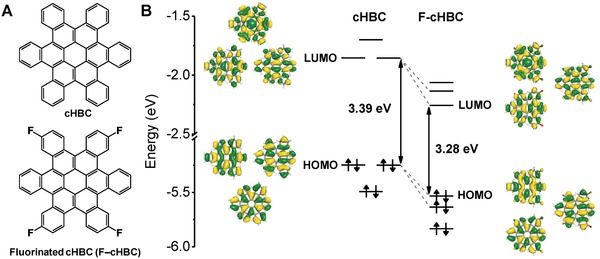
A) Chemical structures and B) DFT‐calculated energy diagrams of the molecular orbitals of contorted hexabenzocoronene (cHBC) and fluorinated cHBC (F—cHBC).

The chemical structures of the cHBC and F—cHBC molecules are shown in **Figure** **1**A. F—cHBC contains four fluorine atoms at its ends that are thought to change the bandgap of the cHBC molecule.^33^, ^34^ The HOMO and LUMO energy levels of F—cHBC estimated using density functional theory (DFT) calculations (HOMO and LUMO energy levels in the Supporting Information) confirmed that fluorine substitution causes two degenerate HOMO and LUMO energy levels to split and decrease from −5.247 to −5.533 eV for the HOMO and −1.856 to −2.257 eV for the LUMO (Figure 1B). The decreased HOMO‐LUMO energy levels of F—cHBC were then blended with a Super P carbon black conducting agent and a poly(vinylidene fluoride) (PVDF) polymer binder (ratio: 80/10/10 wt%) and was subsequently coated onto an 18 µm thick copper current collector and dried overnight at 120 °C in a vacuum oven to expel residual solvent. Tetrahydrofuran (THF) vapor and thermal treatment were used to develop crystal phases and crystallinity of a F—cHBC anode (T‐F—cHBC), which was then assembled into a 2032‐type coin cell.^34^, ^35^ We used 1 m lithium hexafluorophosphate (LiPF_6_) in ethylene carbonate/diethyl carbonate (EC/DEC, ratio: 30/70 vol%) with a fluoroethylene carbonate (FEC, 10 wt%) electrolyte. The fabrication procedure of the crystal‐phase‐controlled T‐F—cHBC electrochemical cell is schematically shown in Figure S1 (Supporting Information).

**Figure 2:**
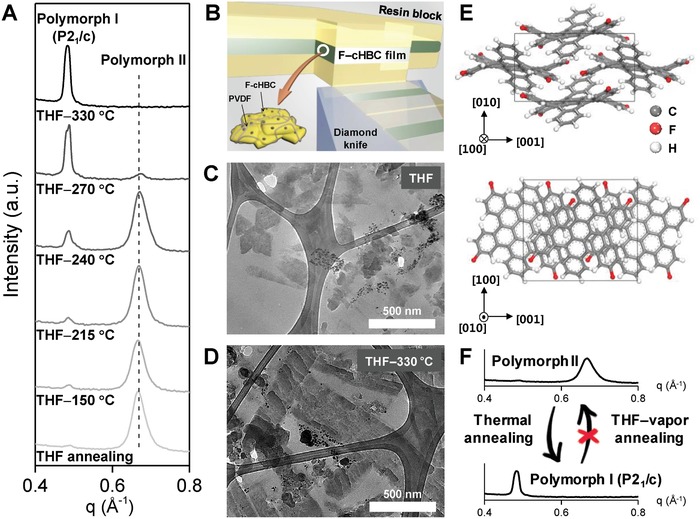
A) 1D GIWAX diffraction traces from in situ GIWAXS as a function of the annealing temperature. B) Schematic illustration of the ultramicrotome method to fabricate ultrathin F—cHBC films. Cross‐sectional TEM image of C) THF‐vapor annealed and D) THF–330 °C annealed F—cHBC films. E) Projection views of the P2_1_/c crystal structure along the [100] (top) and [010] (bottom) directions. Gray: carbon, red: fluorine, white: hydrogen. F) Schematic illustration of phase transformation route of F—cHBC.

To examine the crystal phase evolution of the F—cHBC anode on the current collector, we performed in situ grazing incidence wide‐angle X‐ray scattering (GIWAXS) measurement on the mixtures of F—cHBC/PVDF (ratio: 90/10 wt%) after the samples had been annealed at temperatures from room temperature to 330 °C. The resulting 1D diffraction traces from the in situ GIWAXS measurements showed a monotonic decrease in the peak at *q* = 0.67 Å^−1^ and the formation of a new peak at *q* = 0.49 Å^−1^ as the temperature increased (**Figure** **2**A; Figure S2, Supporting Information). These trends are attributed to a change in the crystal phase of F—cHBC, because a previous report on fluorinated‐cHBC derivatives suggested that a phase change of F—cHBC from polymorph II to polymorph I through thermal treatment is an efficient route for observing the polymorph I phase of polymorphic F—cHBC small molecule.^34^

The detailed microstructures of the F—cHBC samples were further characterized by cross‐sectional transmission electron microscopy (TEM) of ultramicrotomed (50–100 nm) slices of F—cHBC samples (Figure 2B).^32^ The resulting TEM images shown in Figures 2C,D exhibit the distinct characteristics of F—cHBC crystals. For example, the THF‐vapor annealed sample (Figure 2C) had a predominant bright amorphous region with weak selective area electron diffraction (SAED) patterns (Figure S3, Supporting Information), whereas the T‐F—cHBC sample (Figure 2D) showed numerous F—cHBC crystals with strong SAED patterns. The results also suggest that the crystal phase and crystallinity of F—cHBC were affected by the thermal annealing treatment, as the full‐width at half‐maximum (FWHM) of the peak at *q* = 0.49 Å^−1^ for the sample annealed at 330 °C was smaller than that at the *q* = 0.67 Å^−1^ in the sample that was only THF‐annealed (endothermic peak of F—cHBC in differential scanning calorimetry (DSC) analysis; Figure S4, Supporting Information). It is noted that the thermal treatment is not significantly altered the chemical structural change of F—cHBC (Figure S5, Supporting Information). A Monte Carlo computational study with simulated annealing also suggested that polymorph I of the F—cHBC crystal should develop (see the “Crystal structure prediction” section in Supporting Information). The in silico polymorph screening revealed that the experimental X‐ray diffraction (XRD) pattern of the polymorph I phase of F—cHBC matched well with the XRD pattern of the P2_1_/c crystal phase (Figure S6, Supporting Information). Prominent peaks at 7.04°, 12.68°, 14.06°, 14.50°, 16.66°, 18.08°, 18.86°, 21.84°, 22.18°, 23.24°, and 24.10° corresponded to the scattering vectors *q* of 0.50, 0.90, 1.00, 1.03, 1.18, 1.28, 1.34, 1.55, 1.57, 1.64, and 1.70 Å^−1^ and were assigned to the (100), (011), (200), (111), (012), (112), (211), (212), (020), (120), and (12‐1) planes, respectively. Rietveld refinement results suggested the presence of a P2_1_/c crystal phase with lattice parameters of *a* = 12.62 Å, *b* = 8.04 Å, *c* = 14.22 Å, α = 90.00°, β = 89.89°, and γ = 90.00° (Figure 2E). From these results, we can determine the phase transformation processing route of F—cHBC. The produced P2_1_/c crystal phase (polymorph I) could be obtained by thermal annealing but could not be reversibly converted to the polymorph II phase (Figure 2F; Figure S7, Supporting Information). We believe that the obtained crystal phase may be beneficial for facilitating the Li‐ion accessibility in aprotic electrolytes because the previously studied cHBC anodes exhibited enhanced electrochemical performances when the crystals had nanopores in the electrolyte.^32^

### 2.2 Pseudocapacitive Behavior

**Figure 3:**
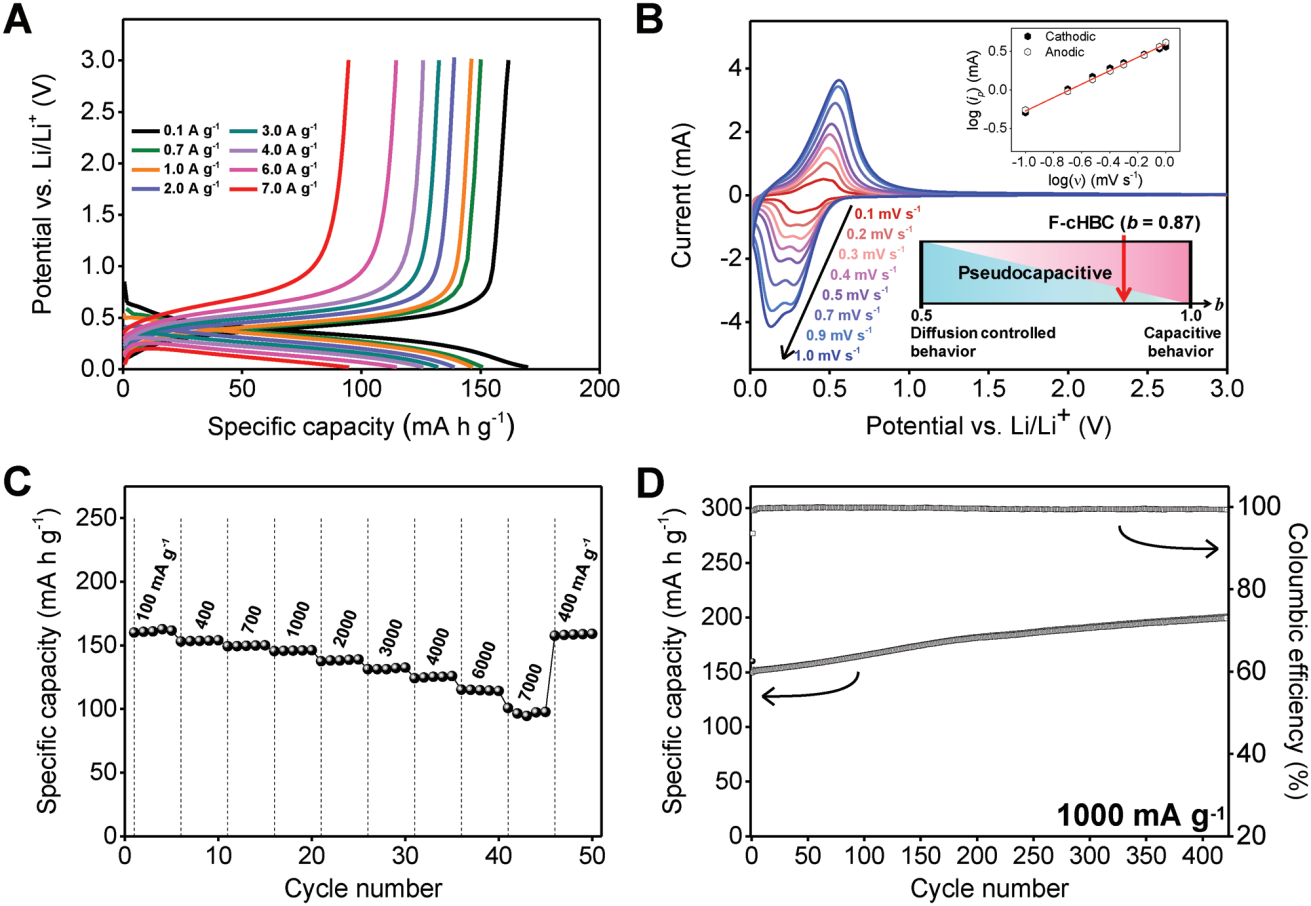
Electrochemical properties of the T‐F—cHBC anode for the Li‐ion storage cell. A) Galvanostatic discharge–charge profiles of the T‐F—cHBC anode from 100 to 7000 mA g^−1^. B) Cyclic voltammograms of the cell containing T‐F—cHBC anode at scan rates from 0.1 to 1.0 mV s^−1^. Upper inset: log–log plot of scan rate (ν) versus peak current (*i*_p_) and regression to estimate *b*. Lower inset: change in contributions of pseudocapacitive behavior. F—cHBC is located in the pseudocapacitive area. C) Rate versus cycle number for the cell with the T‐F—cHBC anode at various current densities from 100 to 7000 mA g^−1^. D) Specific capacity and coulombic efficiency as a function of cycle number at a fixed current density of 1000 mA g^−1^.

We carefully characterized the galvanostatic discharge–charge behavior of the F—cHBC P2_1_/c crystal phase at various current densities (100–7000 mA g^−1^) and voltages (0.02–3.00 V vs Li/Li^+^). As shown in **Figure** **3**A, the voltage profile of the T‐F—cHBC anode dropped continuously until the cutoff potential of 0.02 V and provided a reversible specific capacity of ≈160 mA h g^−1^ at a current density of 100 mA g^−1^. This voltage trajectory and its d*Q* d*V* ^−1^ plot were consistent regardless of the applied current, which contrasts the behavior of conventional Li‐ion intercalation, redox reactions of carbonyl group–based organic electrodes, and previously reported cHBC anode (Figure S8, Supporting Information).^6^, ^32^, ^36^, ^37^ In addition, the recorded cyclic voltammetry (CV) profiles (Figure 3B) showed broad anodic and cathodic peaks, which indicates that the mechanisms of Li‐ion uptake and release differ in the T‐F—cHBC anode, because conventional battery‐like behavior exhibits distinct reduction and oxidation peaks.^13^, ^38^ To confirm this difference, we evaluated the kinetics of the T‐F—cHBC anode by using the equation of *i*_p_ = *av^b^*, where *i*_p_ is the peak current, *v* is the sweep rate, and *a* and *b* are fitted parameters.^13^, ^39^ Parameter *b* can be determined from the slope of the plots of log(*i*_p_) versus log(*v*). When *b* = 1, the capacitive behavior is the dominant process in the cell. When *b* = 0.5, the diffusion‐controlled intercalation process dominates, which is a battery characteristic and has been observed previously in cHBC anode (Figure S9, Supporting Information).^13^, ^32^ In our system, however, *b* was calculated to be 0.87 from the CV curves at a scan rate from 0.1 to 1.0 mV s^−1^. It is noted that the low sweep rate of 1.0 mV s^−1^ was required because the increasing polarization of the reduction peak reaches zero potential. Although value of *b* was close to 1, this intermediate value suggests that the characteristic of the T‐F—cHBC anode is between capacitive and battery characteristics (lower inset of Figure 3B). The quantitative contribution of the capacitive and battery processes can be further quantified by the equation of *i*(*V*) = *k*_1_ *v* + *k*_2_ *v* ^1/2^, where the *k*_1_ *v* represents the contribution of capacitive process, and *k*_2_ *v* ^1/2^ is the contribution of diffusion‐controlled intercalation.^13^, ^40^ The integrated area of stored charge (i.e., capacitance) was 74% of the total integral and indicated that the main reaction of the T‐F—cHBC anode is a capacitance‐dominated pseudocapacitive process and the results further confirmed by Nyquist plot (Figures S10 and S11, Supporting Information).^13^ In addition, the two reduction peaks at ≈0.2 and ≈0.3 V in Figure 3B are expected to be assigned to capacitive and diffusion‐controlled intercalation behaviors because the synchronization difference between the CV curve and shaded region (capacitive behavior) in Figure S12 (Supporting Information) reveals that peak at ≈0.2 V is dominated by a capacitive effect whereas that at ≈0.3 V has contributions from intercalation behavior.

To exploit the pseudocapacitive T‐F—cHBC anode, we performed a set of rate capability tests. As expected, a cell based on the T‐F—cHBC anode maintained a capacity of 100 mA h g^−1^ at a high current density of 7000 mA g^−1^ and delivered a reasonable recovery capacity of 160 mA h g^−1^ at the 42nd cycle (400 mA g^−1^) without significant changes (Figure 3C). In addition, the T‐F—cHBC anode showed a reliable retention capability at a fixed current density of 1000 mA g^−1^. Even at the 400th cycle, the anode had a stable coulombic efficiency of 99.4% and specific capacity of 200 mA h g^−1^ mainly due to the surface redox reaction of the unique pseudocapacitive mechanism. (Figure 3D). It should be noted that the capacity of our cell increased monotonically as the cycle number increased; this trend is presumably due to the contribution of activation sites on the F—cHBC surface during the continuous deep cycling, agreeing with previously reported Li‐ion intercalation‐based cHBC anodes.^32^, ^36^, ^41^

### 2.3 Proposed Li Insertion Mechanism of F—cHBC

**Figure 4:**
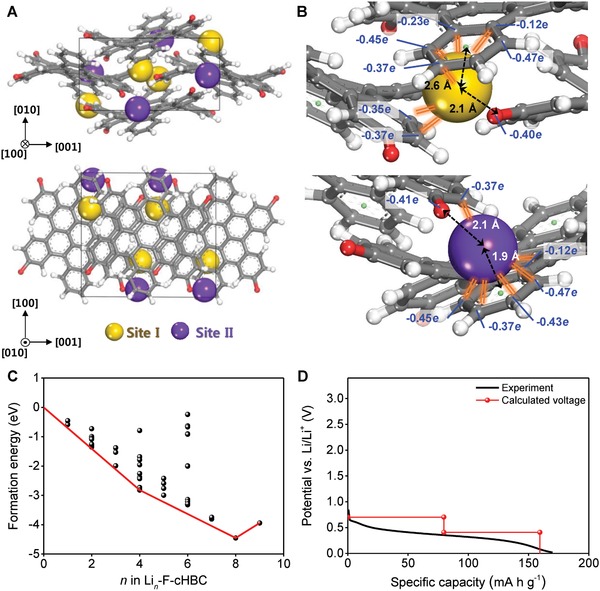
A) Projection views of the optimized P2_1_/c crystal structure of Li‐adsorbed F—cHBC along the [100] (top) and [010] (bottom) directions. Gray: carbon, red: fluorine, white: hydrogen, yellow: lithium at site I, purple: lithium at site II. B) Magnified view of Li‐ion at site I (top) and site II (bottom). Black dotted arrow: distance between Li ion and fluorine or between Li ion and the centroid of the bent edge aromatic ring. Orange line: interaction of adsorbed Li ion with negatively charged atoms. C) Formation energies of Li‐adsorbed F—cHBC as a function of Li‐ion content. Red line: convex hull. D) Experimental (black line) and calculated (red line) voltage profiles.

To identify the adsorption sites of Li ions in the P2_1_/c crystal phase of F—cHBC, we examined the 3D space within the crystal structure by using a probe with a radius of 0.76 Å (i.e., the radius of Li ion) to construct the Connolly surface (Figure S13, Supporting Information). The P2_1_/c crystal structure of F—cHBC has empty spaces with a negative electrostatic potential near the fluorine atoms; these spaces may be capable of accommodating and storing Li ions. To determine whether the empty spaces are active sites that provide energy‐storage capacity, we combined Monte Carlo simulations and DFT calculations (“Monte Carlo simulation” and “Density functional theory calculation” sections in the Supporting Information). The results suggested that these empty spaces with negative electrostatic potentials are the most stable locations for the Li ions (**Figure** **4**A). The empty spaces have two distinct sites, denoted as sites I and II. Crystallographic symmetry yields four identical sites of both types in the unit cell of the P2_1_/c crystal phase. All sites I and II are surrounded by electronegative fluorine atoms and a negatively charged bent edge aromatic ring of the F—cHBC molecule (Figure 4B); the distances between the Li ion and fluorine atom are 2.1 Å at sites I and II, respectively. The distances between the Li ion and the centroid of the bent edge aromatic ring are 2.6 Å at site I and 1.9 Å at site II. For the eight total sites identified in the unit cell, we calculated the formation energy (*E*_F_) of F—cHBC with *n* adsorbed Li ions (Li*_n_*—F—cHBC) as a function of *n* (Figure 4C). The *E*_F_ of Li_4_—F—cHBC was the lowest (i.e., −2.82 eV) when all the Li ions were located at site I; this result suggests that the Li ions are preferentially located at site I rather than site II (Figure S14, Supporting Information). This may be because Li ions are farther from each other at site I than at site II. Also, Li*_n_*—F—cHBC was most thermodynamically stable, with the lowest *E*_F_ of −4.45 eV, when sites I and II were fully occupied by eight Li ions (i.e., Li_8_—F—cHBC). The lowest *E*_F_ for each *n* form an energy convex hull (Figure 4C, red line). The Li*_n_*—F—cHBC begins to become unstable when *n* ≥ 9; i.e., each F—cHBC molecule with four fluorine atoms can store up to four Li ions. This result is in good agreement with the experimental capacity (i.e., ≈160 mA h g^−1^ at a current density of 100 mA g^−1^). Furthermore, the calculated voltage profile is consistent with the experimentally observed continuous voltage drop during Li^+^ storage (Figure 4D); this similarity supports the hypothesis that Li ions are adsorbed at sites I and II near the fluorine atoms.

### 2.4 Capacitive Behavior

**Figure 5:**
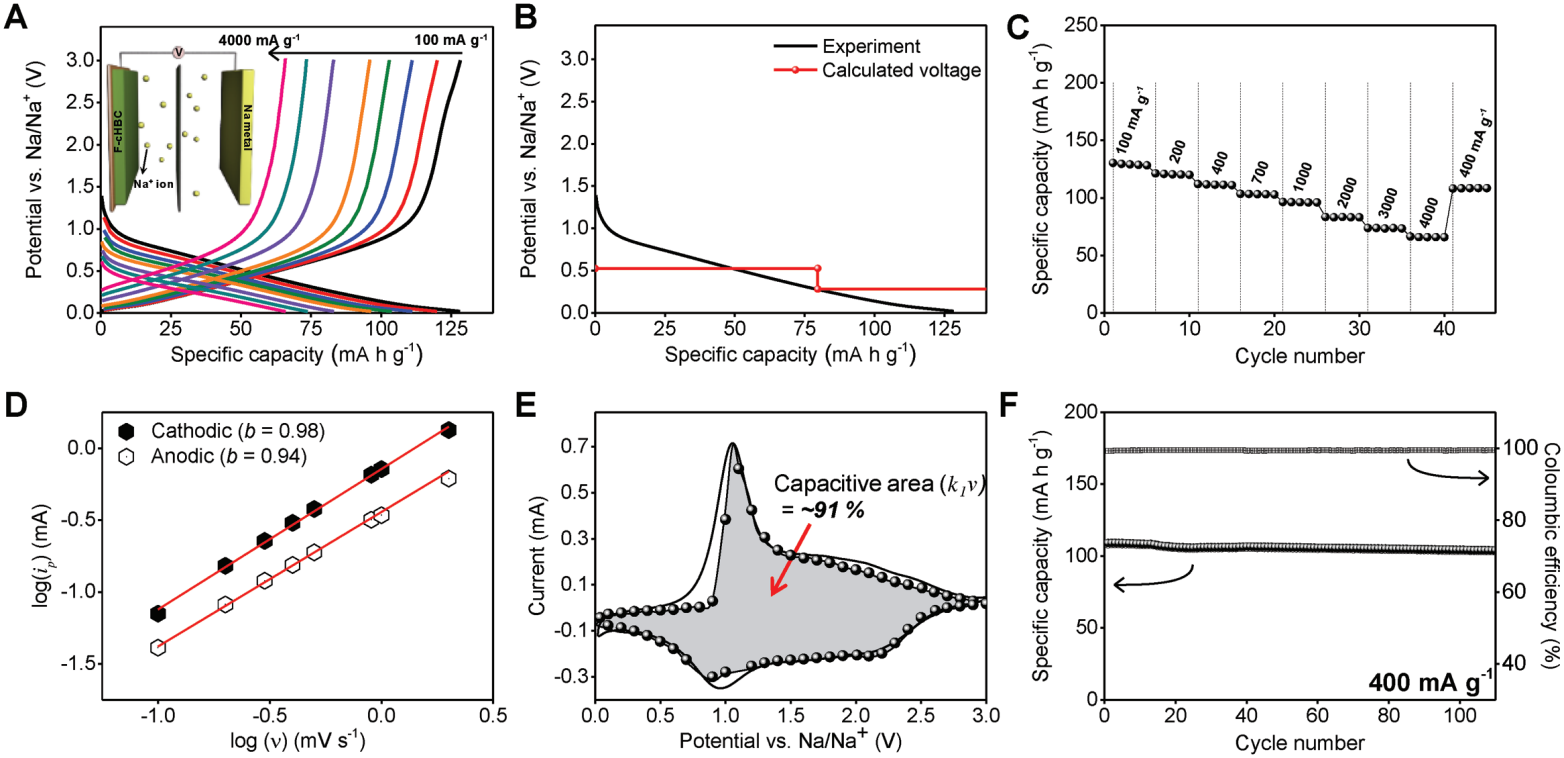
Electrochemical properties of the T‐F—cHBC anode for the Na‐ion storage cell. A) Galvanostatic discharge–charge voltage profiles at various current densities from 100 to 4000 mA h g^−1^. Inset: sodium metal/separator/F—cHBC anode half‐cell structure. B) Experimental (black line) and the calculated (red line) voltage profiles. C) Rate versus cycle number of the Na‐ion cell with the T‐F—cHBC anode ramped from 100 to 4000 mA g^−1^ and back to 400 mA g^−1^. D) Log–log plot of cathodic and anodic peak currents versus sweep rates to calculate *b*. The cathodic *b* = 0.98 and anodic *b* = 0.94. E) CV curve of t T‐F—cHBC electrode at a sweep rate of 1.0 mV s^−1^. Shaded region: capacitive contribution (*k*_1_ν) to total charge storage; ≈91% of the stored charge is by the capacitive process. F) Cycling stability of T‐F—cHBC anode at a constant current density of 400 mA g^−1^.

The T‐F—cHBC anode was also functional in a Na‐ion storage cell. We used the P2_1_/c crystal phase of F—cHBC in a Na‐ion storage cell that was composed of Na metal and 1 m sodium trifluoromethanesulfonate (NaCF_3_SO_3_) in a dimethyl ether (DME) electrolyte. In the galvanostatic measurement shown in **Figure** **5**A, the discharge–charge trajectories revealed that the T‐F—cHBC anode delivers ≈125 mA h g^−1^ specific capacity at a current density of 100 mA g^−1^ (142 mA h g^−1^ at 50 mA g^−1^; Figure S15, Supporting Information). In particular, the DFT calculation for the Na‐ion cell revealed that the adsorption of Na ions at sites I and II is energetically preferred, indicated by the negative *E*_F_ of F—cHBC with the adsorbed Na ions (Figure S16, Supporting Information). As in the Li‐ion case, the Na ions were preferentially located at site I rather than site II. The calculated voltage profile dropped continuously as Na ions were adsorbed; this observation is consistent with the experimental results (Figure 5B). In addition, at a high current density of 4000 mA g^−1^, the T‐F—cHBC anode still delivered 65 mA h g^−1^ and precisely recovered its specific capacity of 108 mA h g^−1^ at 400 mA g^−1^ (Figure 5C). This high specific capacity at a high current rate may be contributed by the pseudocapacitive behavior of F—cHBC. However, the calculated *b*‐value from the CV measurements as a function of scan speed gave *b* ≈ 1, which suggests that the main process of F—cHBC in the Na‐ion cell is to provide capacitor characteristics (Figure 5D; Figure S17, Supporting Information).^13^ The difference in the charge‐storage mechanisms in the Na‐ion cell may be the result of the larger size of Na^+^ compared to that of Li^+^.^40^ The larger diameter of Na^+^ increases the contribution of the surface redox reaction during charge storage, and the resulting process is controlled by capacitance. In addition, the calculated area of the capacitive behavior in the pseudo‐rectangular CV plot was 91%, which is close to the calculated *b*‐value of ≈1 (Figure 5E). Although the F—cHBC exhibited capacitive behavior in the Na‐ion cell, we observed a stable cycling ability with the applied current density of 400 mA g^−1^ (Figure 5F). The cell exhibited a 99.8% coulombic efficiency without significant decay of the specific capacity even after the 110th cycle. Therefore, the results clearly indicate that the presence of fluorine atoms in the P2_1_/c crystal phase of F—cHBC enhances the electrochemical performance, because the cell with pristine cHBC anode showed negligible specific capacity of Na^+^ storage, mainly due to the larger size of the Na ion (Figure S18, Supporting Information). It should be noted that the specific capacity was retained throughout the cycle, which contrasts the behaviors of F—cHBC and previously reported cHBC‐based Li‐ion cells. One of the reasons is that the capacitive‐dominant behavior of F—cHBC in the Na‐ion cell effectively prevents crystal alterations during the discharge–charge processes, resulting in limited growth of activation sites in the F—cHBC anode (Figure S19, Supporting Information).

## 3 Conclusion

By fluorinating the cHBC molecule, we achieved capacitive characteristics in a small organic molecule for Li‐ and Na‐ion storage cells. The F—cHBC anode with a controlled crystal phase in the Li‐ion cell could operate for more than 400 cycles without changes of its performance and provided an adequate specific capacity of 100 mA h g^−1^ at a high current density of 7000 mA g^−1^. DFT calculations showed that adsorption of Li and Na ions is energetically favorable in the empty space between the fluorine atom of the F—cHBC molecule and the negatively charged bent aromatic ring. Furthermore, F—cHBC electrodes can also be used in Na‐ion storage cells. The high specific capacity of 65 mA h g^−1^ obtained at 4000 mA g^−1^ may result from the capacitive behavior of the F—cHBC anode in the Na‐ion cell. More importantly, there are a few materials that can be used in both Li‐ and Na‐ion storage cells. Thus, this unique electrochemical behavior of F—cHBC can provide a new way to develop electrochemical organic capacitive electrodes for alkali‐ion storage cells.

## 4 Experimental Section

*Synthesis of F*—*cHBC and Preparation of* ***T‐F—cHBC*** *Electrode*: F—cHBC was synthesized following the same procedure used for synthesizing cHBC (see the Supporting Information for details). The specific surface area of F—cHBC powder is 28.3 m^2^ g^−1^ (Figure S20, Supporting Information). To prepare the F—cHBC working electrode, the slurry (F—cHBC active material), carbon black (super P, TIMCAL) conducting agent, and PVDF binder (ratio: 80/10/10 wt%) in a *N*‐methyl‐2‐pyrrolidone (NMP) solvent) were spread onto an 18 µm thick copper foil using a doctor blade (Wellcos Co., Korea), which was subsequently dried at 120 °C in a vacuum oven overnight to eliminate any residual solvent and moisture. The mass loading of the electrodes was ≈1.5 mg cm^−2^. For the THF annealing; a piece of the F—cHBC electrode was placed in the closed 80 mL vial with saturated THF (vapor pressure at 20 °C: 143 mmHg) for 4 h. The thermal annealing of the F—cHBC electrode was performed at 330 °C in an Ar‐filled tube furnace for 30 min and slowly cooled to room temperature.^34^, ^35^

*Characterization*: F—cHBC powder was characterized by using XRD (Rigaku D/MAX25000V/PC power diffractometer, Cu Kα radiation, λ = 1.54178 Å) and DSC (Q20, TA instruments) with a heating ratio of 5 °C min^−1^. Morphology analysis of F—cHBC was performed using TEM (JEM‐1400). To prepare the ultrathin F—cHBC film, the F—cHBC film was submerged in an epoxy resin using a block mold, after which the cured resin block was sectioned into 50–100 nm thick slabs using an ultramicrotome (RMC CR–X) with a diamond knife.^32^ The polymorphic analysis of F—cHBC was performed by GIWAXS on the 6D UNIST–PAL line at the Pohang Accelerator Laboratory (Pohang University of Science and Technology, Korea). GIWAXS samples were prepared by spreading the slurry containing the F—cHBC and PVDF binder (ratio: 90/10 wt%) onto Si wafers using a doctor blade. The F—cHBC sample was irradiated with monochromatized X‐rays (λ = 1.06879 Å) with a fixed grazing incidence angle of 0.13°. In situ GIWAXS tracing was conducted as a function of thermal annealing temperature.

For the Li‐ion storage electrochemical analysis, the T‐F—cHBC working electrodes were assembled in 2032‐type coin cells containing 300 µm Li‐foil counter electrodes, polyethylene film separators (Celgard 2400), and 1 m LiPF_6_ in EC/DEC (ratio: 30/70 vol%) with 10 wt% FEC electrolyte. For Na‐ion storage electrochemical characterization, a sodium‐metal foil counter electrode, quartz separator (Whatman, QMA grade), and 1 m sodium NaCF_3_SO_3_ in a DME electrolyte were assembled in a 2032‐type coin cell. Galvanostatic discharge–charge rate capability tests were performed using a battery tester (Wonatech, WBCS‐3000L) between 0.02 and 3.00 V versus Li/Li^+^ or Na/Na^+^. CV measurements were performed using multichannel potentiostats (BioLogic/VSP‐300) at various scan rates.

## Conflict of Interest

The authors declare no conflict of interest.

## Supporting information

Supplementary
