# Supplementary material for: Capacitive Organic Anode Based on Fluorinated‐Contorted Hexabenzocoronene: Applicable to Lithium‐Ion and Sodium‐Ion Storage Cells
Source: Adv Sci (Weinh). 2018 Nov 2;5(12):1801365. doi: 10.1002/advs.201801365 (PMC6299712; doi:10.1002/advs.201801365)
Supplement: Supplementary file 1 — Supplementary [file ADVS-5-1801365-s001.pdf]

Copyright WILEY-VCH Verlag GmbH & Co. KGaA, 69469 Weinheim, Germany, 2018.

Supporting Information

for *Adv. Sci.,* DOI: 10.1002/advs.201801365

Capacitive Organic Anode Based on Fluorinated-Contorted Hexabenzocoronene: Applicable to Lithium-Ion and Sodium-Ion Storage Cells

Jaehyun Park, Cheol Woo Lee, Ju Hyun Park, Se Hun Joo, Sang Kyu Kwak, Seokhoon Ahn,* and Seok Ju Kang**

Copyright WILEY-VCH Verlag GmbH & Co. KGaA, 69469 Weinheim, Germany, 2018.

Supporting Information

Capacitive Organic Anode Based on Fluorinated-Contorted Hexabenzocoronene: Applicable to Lithium-Ion and Sodium-Ion Storage Cells

*Jaehyun Park, Cheol Woo Lee, Ju Hyun Park, Se Hun Joo, Sang Kyu Kwak**, Seokhoon Ahn**, and Seok Ju Kang**

Keywords: contorted hexabenzocoronene, fluorination, pseudocapacitor, electrochemical capacitor, Li-ion cell, Na-ion cell

Experimental section

Synthesis of fluorinated contorted hexabenzocoronene (F–cHBC)

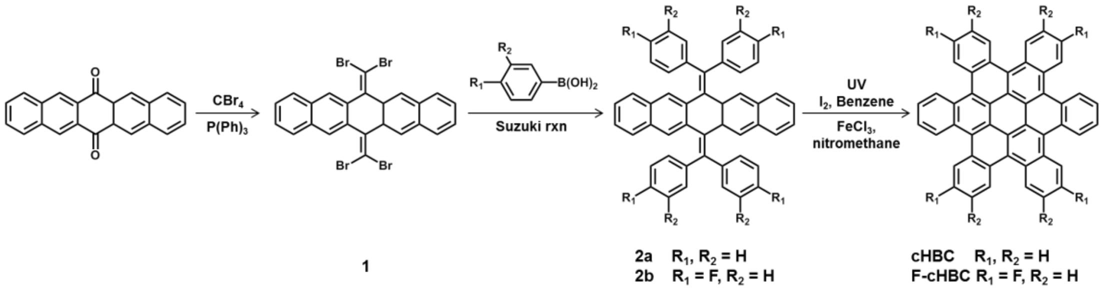

Scheme S1. Synthetic scheme of fluorinated contorted hexabenzocoronene (F–cHBC)

The cHBC and F–cHBC molecule containing four fluorine atoms at the end of cHBC were synthesized by following a procedure reported previously (**Scheme S1**).[1–4]

6,13-bis(bis(dibromomethylene)-6,13-dihydropentacene (1). In a 500 ml 1 neck roundbottom flask were added 6,13-pentacenedione (10.0 g, 32.43 mmol), tetrabromomethane (32.25 g, 97.29 mmol), triphenylphosphine (51.04 g, 194.58 mmol) and 300 ml of toluene. The resulting mixture was stirred and refluxed for 24 h at 80℃. The mixture was filtered to remove the solid and the filtrate was evaporated under reduced pressure. The resulting solid was recrystallized by dichloromethane and methanol. The white solid of 6.0 g was obtained

and the yield was 30.01% ^{1}H NMR. (600MHz, $CDCl_3$, ppm) δ 8.28(s, 4H), 7.85(m, 4H), 7.51(m, 4H)

6,13-bis(bis(4-fluorophenyl)methylene)-6,13-dihydropentacene (2b). 1 (3.0 g, 4.84 mmol), 4-fluorophenylboronic acid (4.06 g, 29.034 mmol), potassium phosphate tribasic (12.34g, 58.067 mmol) and tetrakis(triphenylphosphine)-palladium(0) (1.12 g, 0.968 mmol) were dissolved in 250 ml of 1,4-dioxane in a 500 ml roundbottom flask. The solution was stirred and refluxed for 24 h at 100℃. The resulting solution was extracted with deionized water and ethyl acetate. The organic layer was dried with $MgSO_4$ and then the solvent was removed by using rotary evaporator. The remained solid was recrystallized by dichloromethane and methanol and then purified by column chromatography using hexane and dichloromethane (5:1). The white solid was obtained and the yield was 1.9 g (57.33%) ^{1}H NMR. (600MHz, $CDCl_3$, ppm) δ 7.488(s, 4H), 7.42(m, 2H), 7.39(t, J = 6.1Hz, 8H), 7.28(s, 4H), 6.98(t, J = 7.7Hz, 8H) MS (MALDI): cald. for $C_{48}H_{28}F_4$: 680.21 found: 680.06

Tetrafluorinated contorted hexabenzocoronene (F–cHBC). A mixture of **2b** (0.40 g, 0.588 mmol), iodine (0.37 g, 1.47 mmol) and propylene oxide (30 ml) in 200 ml of benzene were irradiated with UV light at room temperature. After 24 hours of irradiation, the solvent was removed by rotary evaporator. In a 500 ml round bottom flask were added the resulting solid (0.40 g, 0.588 mmol), iron(III) chloride (0.763 g, 4.704 mmol), nitromethane (20 ml) and dichloromethane (20 ml). The mixture was stirred under a nitrogen atmosphere for 24h at room temperature. The solvent was removed by rotary evaporator and then the remained solid was recrystallized by dichloromethane and methanol. The solid was filtered and then washed with dichloromethane (0.30g, 76%). ^{1}H NMR (600MHz, $CDCl_3$, ppm) δ 9.22(s, 4H), 9.15(s, 4H), 8.91(s, 4H), 7.89(s, 4H), 7.60(s, 4H) MS (MALDI): cald. for $C_{48}H_{20}F_4$: 672.15 found: 672.28

Computational section

HOMO and LUMO energy levels

To estimate the highest occupied molecular orbital (HOMO) and the lowest unoccupied molecular orbital (LUMO) energy levels of cHBC and F–cHBC molecules, spin-polarized density functional theory (DFT) calculations were conducted using the DMol3 module[5,6] of the Materials Studio 2018.[7] The Becke's three-parameter hybrid exchange functional combined with the Lee-Yang-Parr correlation functional (B3LYP)[8,9] was employed for describing the exchange-correlation potential of electrons. The DNP 4.4 basis set was used with a global orbital cutoff of 3.7 Å. The core electrons were explicitly treated as all electrons with relativistic effect. The long-range van der Waals interactions were corrected using the Grimme's method.[10] The self-consistent field calculation was performed with the fixed orbital occupancy, until the convergence criterion of 1.0×10^{-6} was satisfied. The convergence criteria for geometry optimization were set to 1.0×10^{-5} Ha for the maximum energy change, 0.002 Ha $Å^{-1}$ for the maximum force, and 0.005 Å for the maximum displacement, respectively.

Crystal structure prediction

The computational polymorphism study of F–cHBC was carried out using the Polymorph module of the Materials Studio 2018.[7] Using the optimized structure of F–cHBC molecule as input, the ab initio prediction of polymorphs was performed sequentially in six steps (i.e., packing, clustering, geometry optimization, clustering, geometry optimization, and clustering). In the packing step, the crystal structures belonging to a specific space group were sampled using Monte Carlo simulated annealing. The packing procedure was performed for 10 different space groups: $P2_1/c$, $P\bar{1}$, $P2_12_12_1$, $C2/c$, $P2_1$, $Pbca$, $Pna2_1$, Cc, $Pbcn$, and $C2$. To achieve sufficiently wide sampling, we set the maximum temperature to 1.5×10^5 K, the minimum temperature to 300 K, the maximum number of steps to 500,000, the number of steps to accept before cooling to 100, the minimum move factor to 1.0×10^{-50}, and the

heating factor to 0.025. In the geometry optimization step, the lattice parameters and atomic positions were relaxed under crystallographic symmetry. The F–cHBC molecule was treated rigid body in the first geometry optimization step, while it was fully relaxed in the second geometry optimization step. The maximum number of steps was set to 10,000 and the convergence criteria were set to 2.0×10^{-5} kcal mol^{-1} for the maximum energy change, 0.001 kcal mol^{-1} $Å^{-1}$ for the maximum force, 0.001 GPa for the maximum stress, and 1.0×10^{-5} Å for the maximum displacement. In the clustering step, many similar structures were grouped into clusters, and the lowest energy structure representing each cluster was filtered. The criterion of crystal similarity measure was set to 0.11, which was calculated based on a comparison of radial distribution functions with a cutoff distance of 7 Å and 140 bins. After the final clustering step, the space group symmetry of the predicted crystal structures was reanalyzed and the in-silico screening was carried out on the basis of XRD comparison. The interatomic interactions were described by COMPASS II force field[11] and calculated using the Ewald summation method.[12,13]

Monte Carlo simulation

To figure out the specific adsorption sites of Li-ions in the crystal structure of F–cHBC, Monte Carlo simulated annealing was performed using the Sorption module of Materials Studio 2018.[7] Based on the metropolis algorithm, the Monte Carlo simulated annealing was carried out with the maximum number of loading steps of 1.0×10^{5}, the maximum number of production steps of 1.0×10^{8}, and 40 annealing cycles. All simulations were repeated 5 times independently. The interatomic interactions were described by COMPASS II force field[11] with Mulliken[14] charges obtained by DFT calculations.

Density functional theory calculation

DFT calculations were performed using the CASTEP module[15] of the Materials Studio 2018.[7] The generalized gradient approximation with the Perdew–Burke–Ernzerhof (GGA-PBE) functional[16] was used to describe the exchange correlation potential of the electrons. The interactions between ions and electrons were described by on-the-fly generated norm-conserving pseudopotentials. The plane-wave basis set with a cutoff energy of 840 eV was employed to expand the wave functions. The van der Waals interactions were corrected using Grimme's method.[10] The convergence criterion for self-consistent field calculation was set to 5.0×10^{-7} eV atom^{-1}. Lattice parameters and atomic positions were fully relaxed. The convergence criteria for geometry optimization were set to 5.0×10^{-6} eV atom^{-1} for energy, 0.01 eV Å^{-1} for force, 0.02 GPa for stress, and 5.0×10^{-4} Å for displacement. The Brillouin zone was integrated using a $1 \times 2 \times 1$ k-point grid with the Monkhorst–Pack scheme[17] for all calculations. The formation energy (E_f) of the inserted structure as a function of Li-ion content was calculated as follows:

$$E_{\mathrm{f}} = E_{\mathrm{Li}_n\text{-F-cHBC}} - E_{\mathrm{F-cHBC}} - nE_{\mathrm{Li}}$$

where $E_{\mathrm{Li}_n\text{-F-cHBC}}$ is the total energy of the F–cHBC crystal with adsorbed Li-ions, n is the number of adsorbed Li-ions, $E_{\mathrm{F-cHBC}}$ is the total energy of the $P2_1/c$ crystal phase of F–cHBC, and E_{Li} is the total energy per atom of bcc bulk Li. The voltage profile as a function of Li-ion content was calculated as follows:

$$V(n) = -\frac{E_{\mathrm{Li}_{n_2}\text{-F-cHBC}} - E_{\mathrm{Li}_{n_1}\text{-F-cHBC}} - (n_2 - n_1)E_{\mathrm{Li}}}{q(n_2 - n_1)}.$$

where $E_{\mathrm{Li}_{n_1}\text{-F-cHBC}}$ and $E_{\mathrm{Li}_{n_2}\text{-F-cHBC}}$ represent the total energy of the F–cHBC crystal with inserted Li-ions, n_1 and n_2 are the numbers of adsorbed Li-ions ($n_2 > n_1$), E_{Li} is the total energy per atom of bcc bulk Li, and q is the net charge of Li-ions ($q = +1e$).

References

[1] S. Xiao, M. Myers, Q. Miao, S. Sanaur, K. Pang, M. L. Steigerwald, C. Nuckolls, *Angew. Chem. Int. Ed.* **2005**, *44*, 7390−7394.
[2] S. Xiao, J. Tang, T. Beetz, X. Guo, N. Tremblay, T. Siegrist, Y. Zhu, M. Steigerwald, C. Nuckolls, *J. Am. Chem. Soc*. **2006**, *128*, 10700−10701.
[3] C.-Y. Chiu, B. Kim, A. A. Gorodetsky, W. Sattler, S. Wei, A. Sattler, M. Steigerwald, C. Nuckolls, *Chem. Sci.* **2011**, *2*, 1480−1486.
[4] J. Park, C. W. Lee, S. H. Joo, J. H. Park, C. Hwang, H.-K. Song, Y. S. Park, S. K. Kwak, S. Ahn, S. J. Kang, *J. Mater. Chem. A* **2018**, *6*, 12589–12597.
[5] B. Delley, *J. Chem. Phys.* **2000**, *113*, 7756–7764.
[6] B. Delley, *J. Chem. Phys.* **1990**, *92*, 508–517.
[7] Dassault Systèmes BIOVIA, Materials Studio, **2018**, San Diego: Dassault Systèmes, 2018.
[8] A. D. Becke, *J. Chem. Phys.* **1993**, *98*, 5648–5652.
[9] P. J. Stephens, F. J. Devlin, C. F. Chabalowski and M. J. Frisch, *J. Phys. Chem.* **1994**, *98*, 11623–11627
[10] S. Grimme, *J. Comput. Chem.* **2006**, *27*, 1787–1799.
[11] H. Sun, Z. Jin, C. Yang, R. L. Akkermans, S. H. Robertson, N. A. Spenley, S. Miller, and S. M. Todd, *J. Mol. Model* **2016**, *22*, 47.
[12] P. P. Ewald, *Annalen der Physik* **1921**, *369*, 253–287.
[13] M. P. Tosi, in Solid State Physics, eds. F. Seitz and D. Turnbull, Academic Press, **1964**, *16*, pp. 1–120.
[14] R. S. Mulliken, *J. Chem. Phys.* **1955**, *23*, 1833–1840.
[15] S. J. Clark, M. D. Segall, C. J. Pickard, P. J. Hasnip, M. J. Probert, K. Refson, and M. C. Payne, *Zeitschrift für Kristallographie* **2005**, *220*, 567–570.
[16] J. P. Perdew, K. Burke and M. Ernzerhof, *Phys. Rev. Lett.* **1996**, *77*, 3865.
[17] H. J. Monkhorst and J. D. Pack, *Phys. Rev. B* **1976**, *13*, 5188–5192.

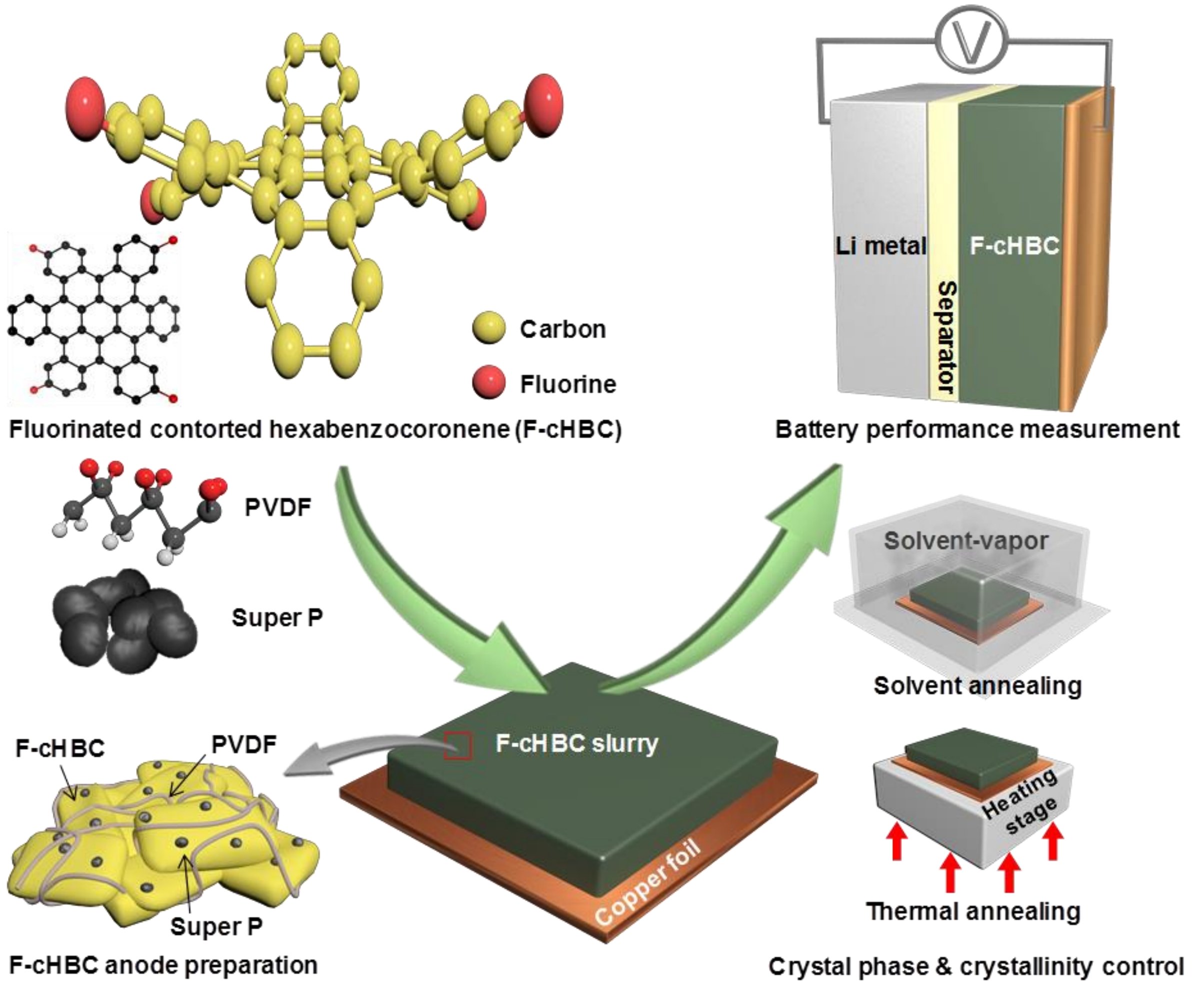

Figure S1. Schematic illustration of the summarized process of fabricating F–cHBC electrode for alkali- ion storage cells.

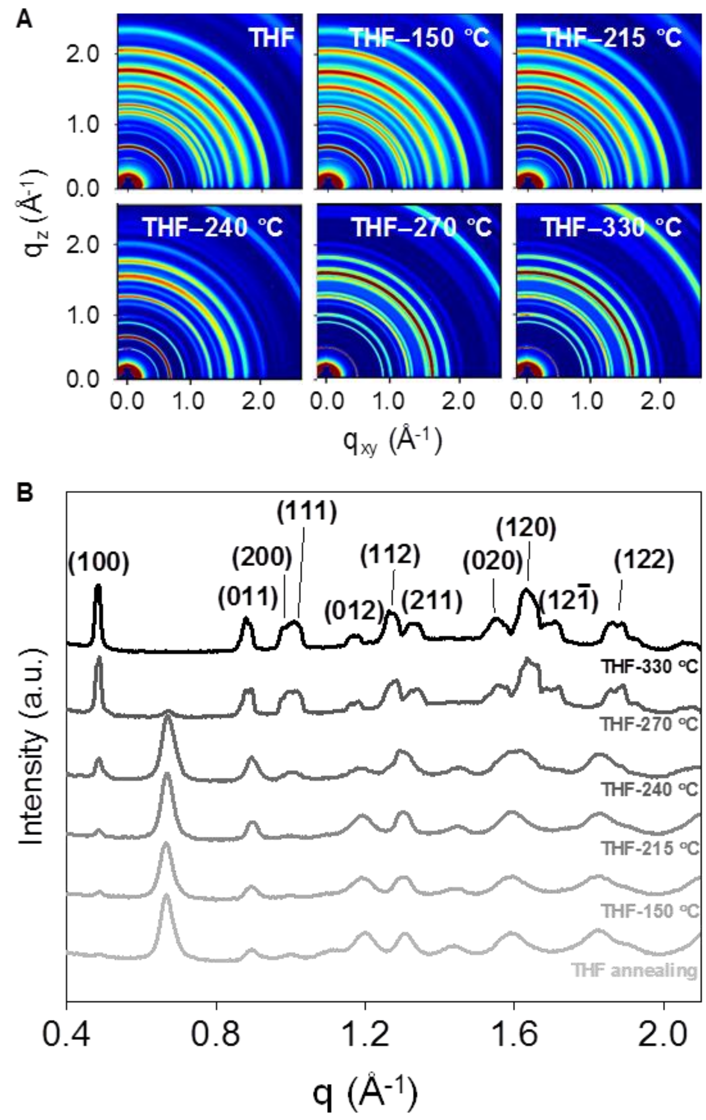

Figure S2. A) *in situ* 2D-GIWAXS images and B) 1D diffraction traces of THF–vapor annealed F–cHBC film on a Si wafer.

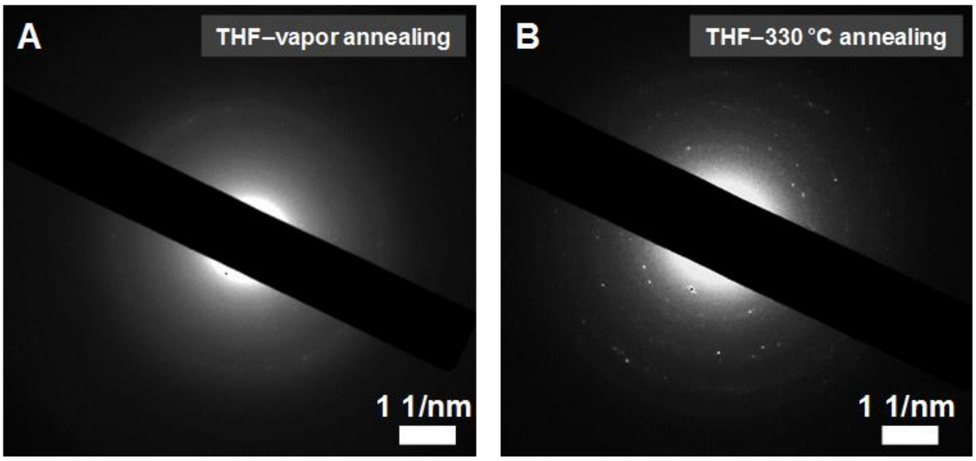

Figure S3. Selected area electron diffraction (SAED) patterns or the A) THF–vapor annealed and B) THF–330 °C annealed F–cHBC film.

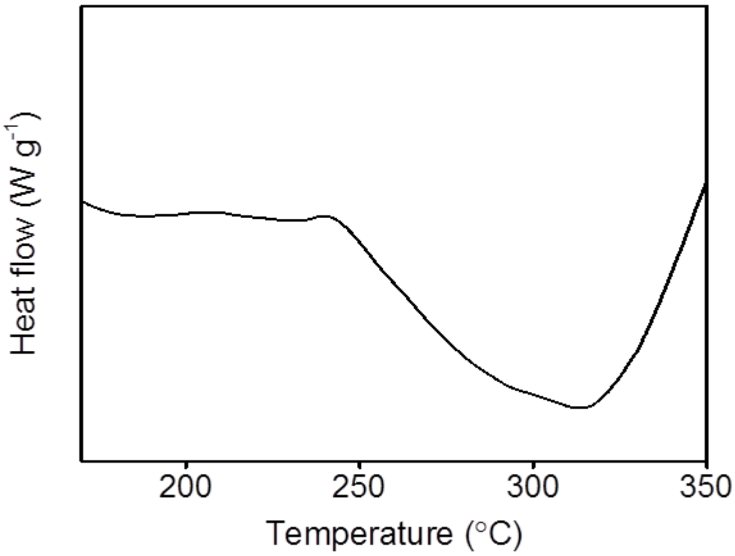

Figure S4. Diffraction scanning calorimetry (DSC) analysis of F–cHBC powder with scan rate of 5 °C min^{-1}.

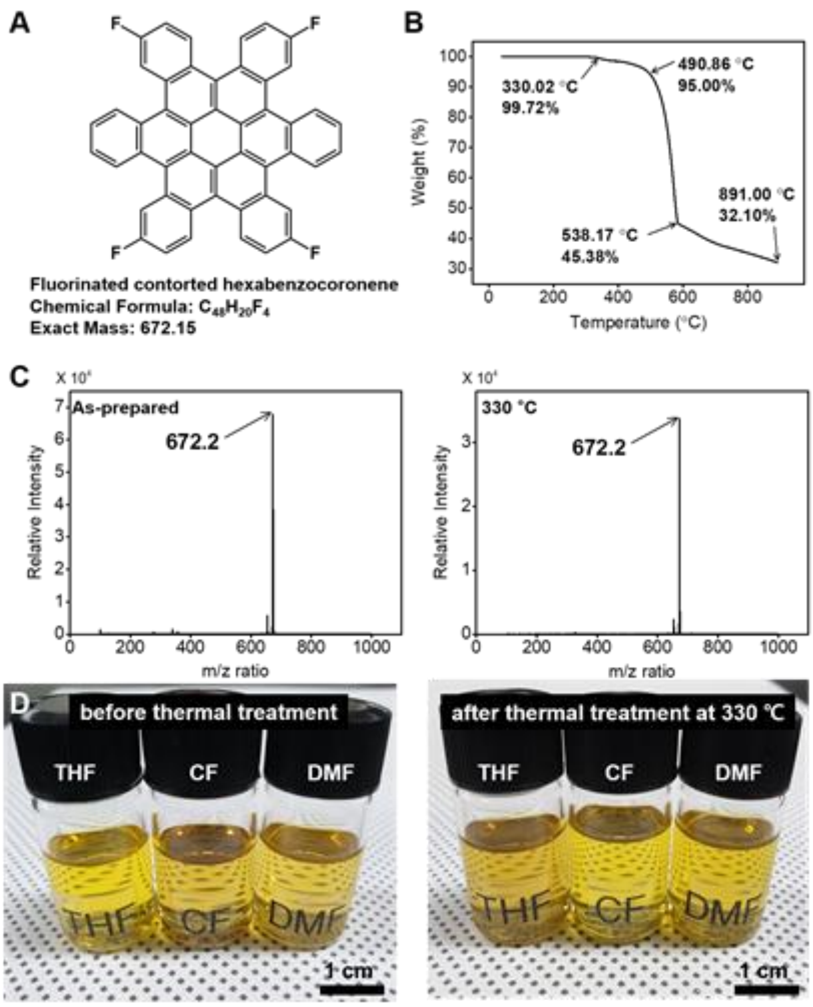

Figure S5. A) Chemical structure of F–cHBC, B) TGA curve of F–cHBC, C) mass spectrum of F–cHBC after annealing at room temperature and 330 °C obtained using laser desorption/ionization time-of-flight mass spectrometry, and D) solubility test of F–cHBC after annealing at 330 °C.

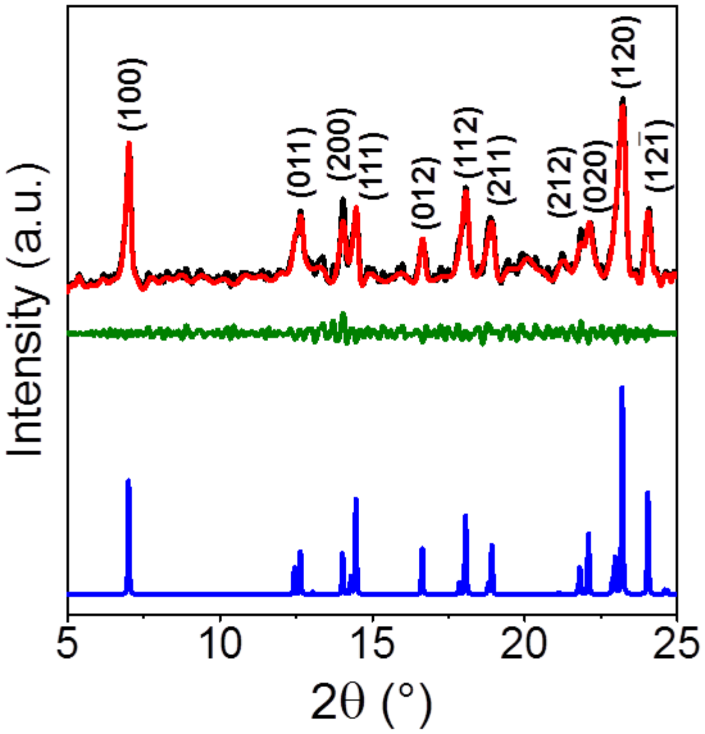

Figure S6. XRD patterns of F–cHBC: experimental (black line), Rietveld refined (red line), their difference (green line), and the $P2_1/c$ crystal phase (blue line). (R_{wp}= 13.21 %, R_p= 9.74 %)

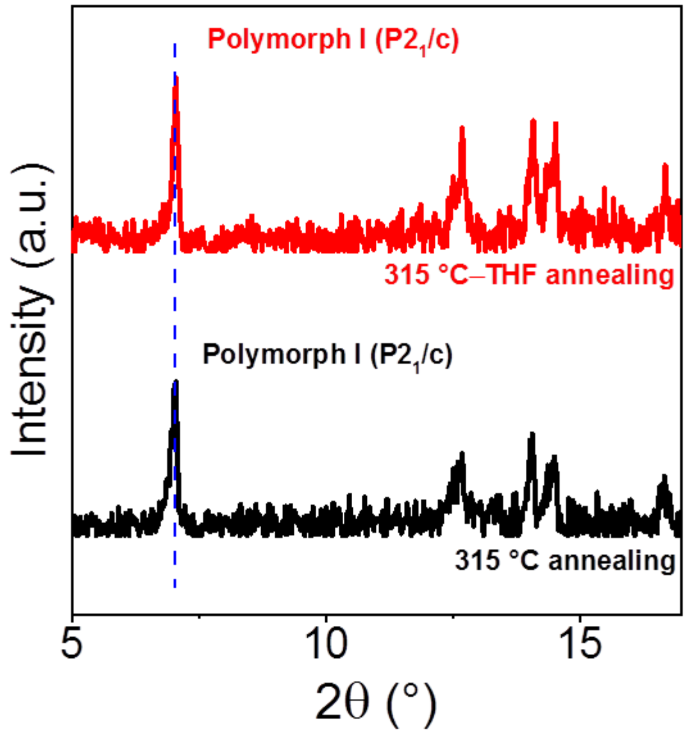

Figure S7. X-ray diffraction patterns of 315 °C annealed (black line) and 315 °C–THF-vapor annealed F–cHBC (red line).

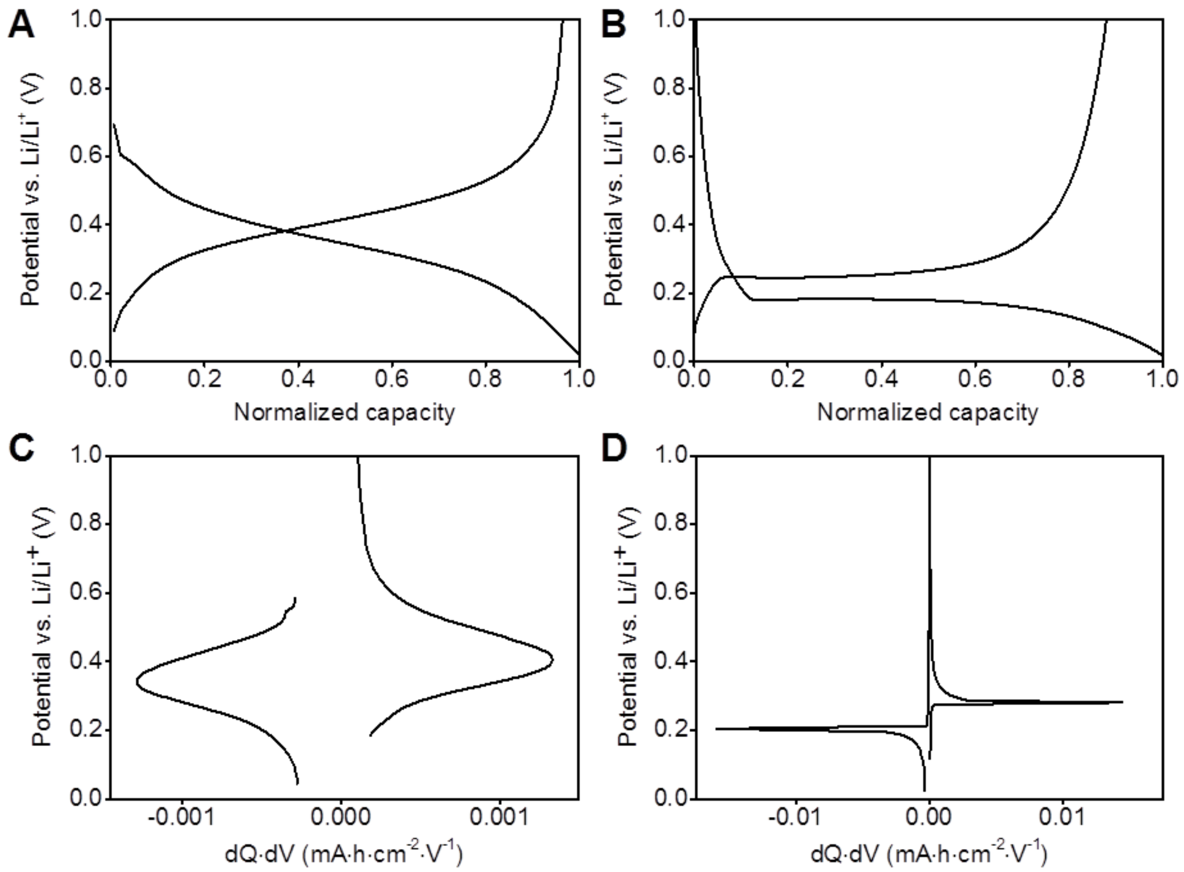

Figure S8. Galvanostatic discharge–charge profiles of A) T-F–cHBC and B) cHBC anodes contained in Li-ion cells. Corresponding differential capacity curves of C) T-F–cHBC and D) cHBC anodes.

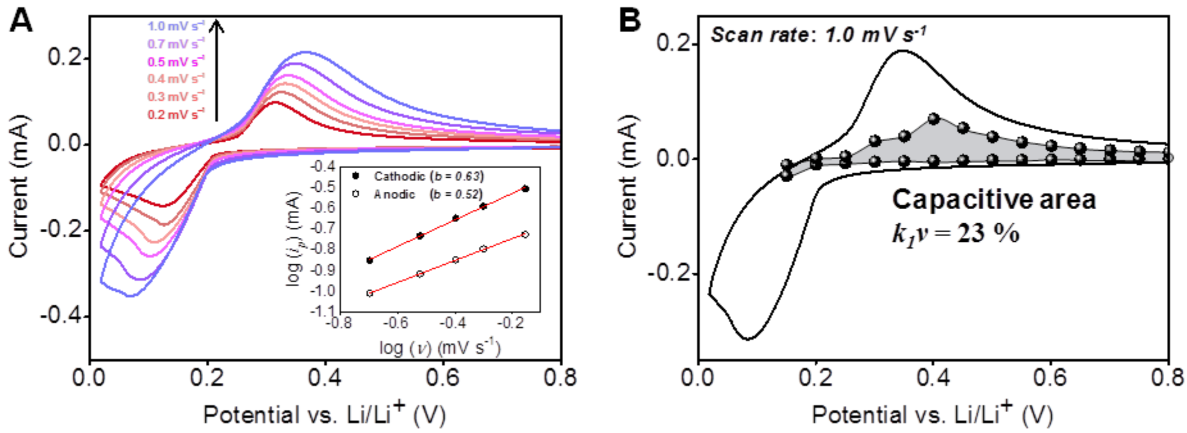

Figure S9. A) CV curves of the Li-ion cell containing the cHBC anode at scan rates from 0.2 to 1.0 mV s^{-1}. Lower inset shows the *log-log* plot of scan rate (v) versus peak current (i_p). B) CV curve at 1.0 mV s^{-1}. Shaded region represented the capacitive contribution (k_1v) to total charge storage.

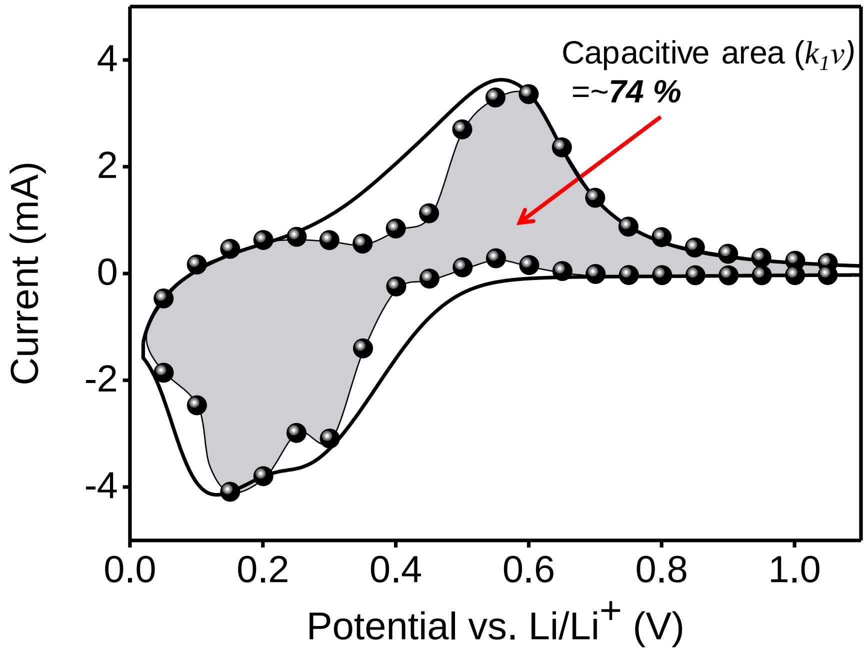

Figure S10. Cyclic voltammogram of F–cHBC electrode at a scan rate of 1 mV s^{-1} (Li-ion storage cell). The current of capacitive behavior (k_1v) at certain voltages are plotted on the CV curve.

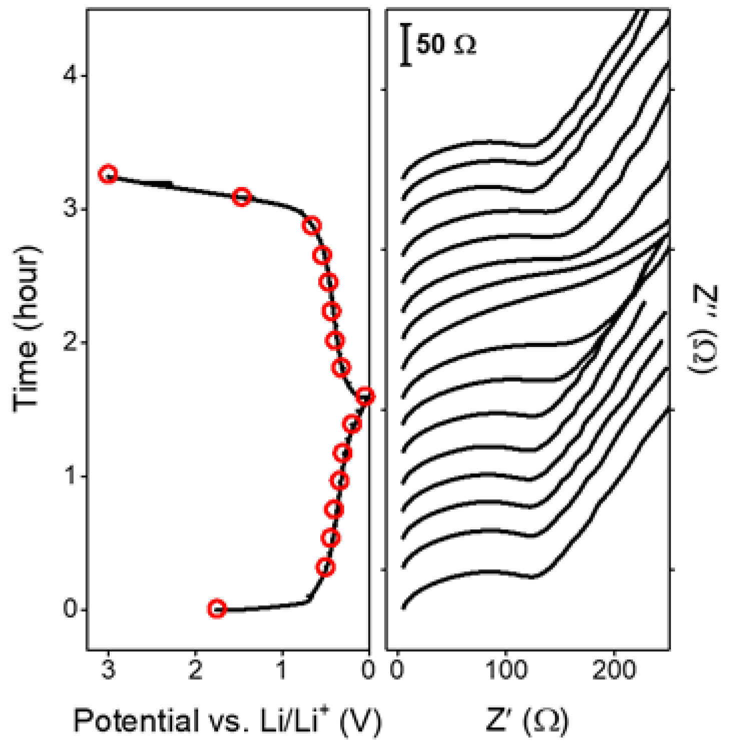

Figure S11. Nyquist plots of F–cHBC contained Li-ion cell according to the discharge-charge cycle at a fixed current density of 0.1 A g^{-1}.

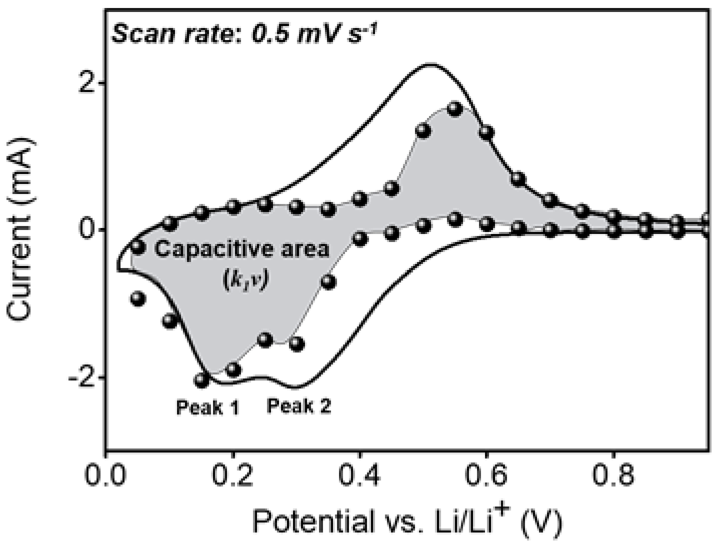

Figure S12. CV curve of the F–cHBC anode at a sweep rate of 0.5 mV s^{-1}. The shaded region indicates the capacitive contribution (k_1v) to the total charge storage.

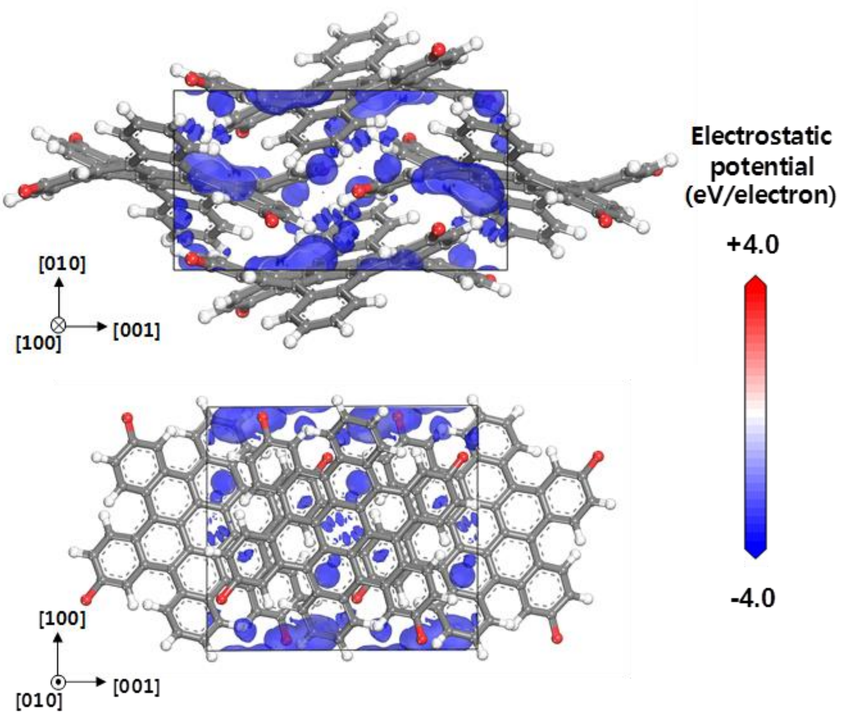

Figure S13. The optimized structure and electrostatic potential maps of F–cHBC. The electrostatic potential is mapped onto the Connolly surface. Carbon, hydrogen, and fluorine are colored grey, white, and red, respectively

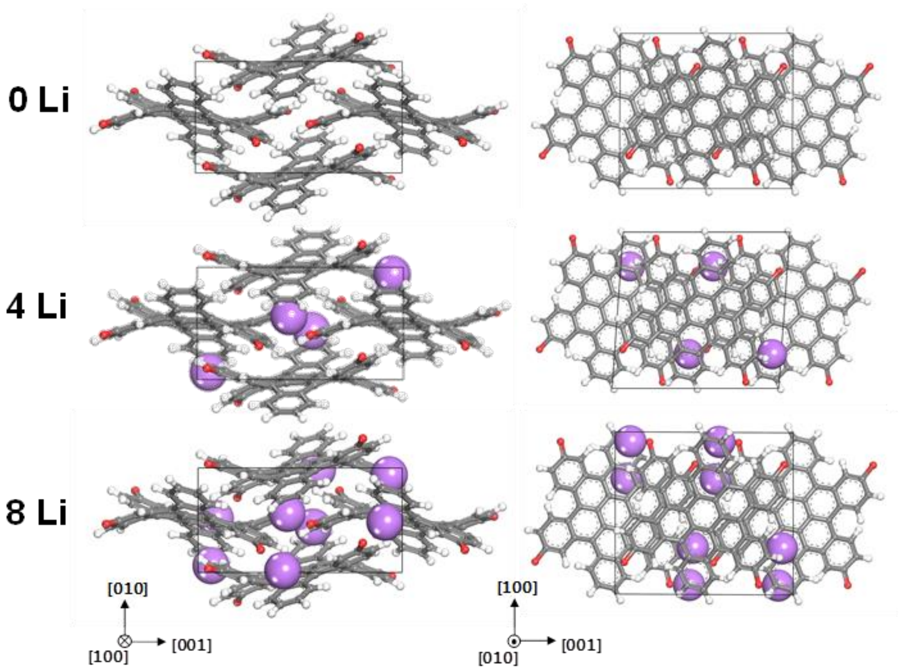

Figure S14. Projection views of the optimized $P2_1/c$ crystal phase and 4, 8 Li-adsorbed F–cHBC stable structures along the [100] and [010] directions. Carbon, hydrogen, fluorine and lithium are colored grey, white, red and purple, respectively.

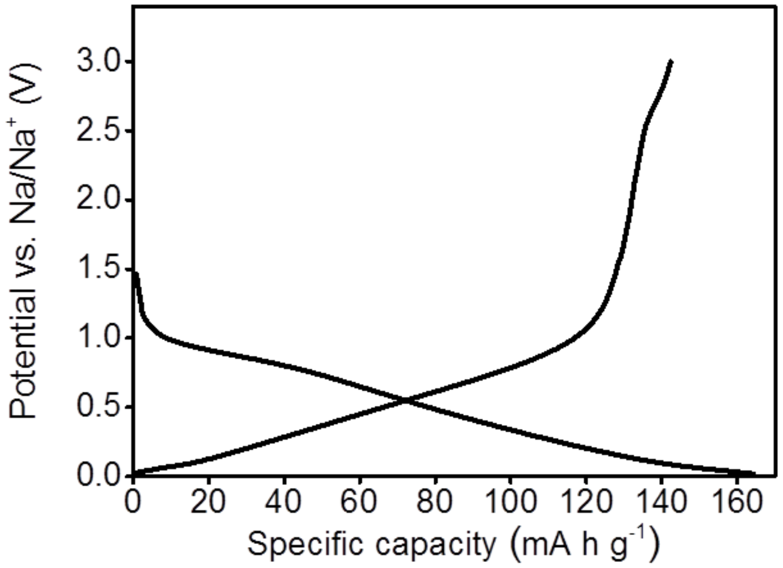

Figure S15. Galvanostatic discharge-charge voltage profile of F–cHBC in Na-ion storage cell at fixed current density of 50 mA g^{-1}.

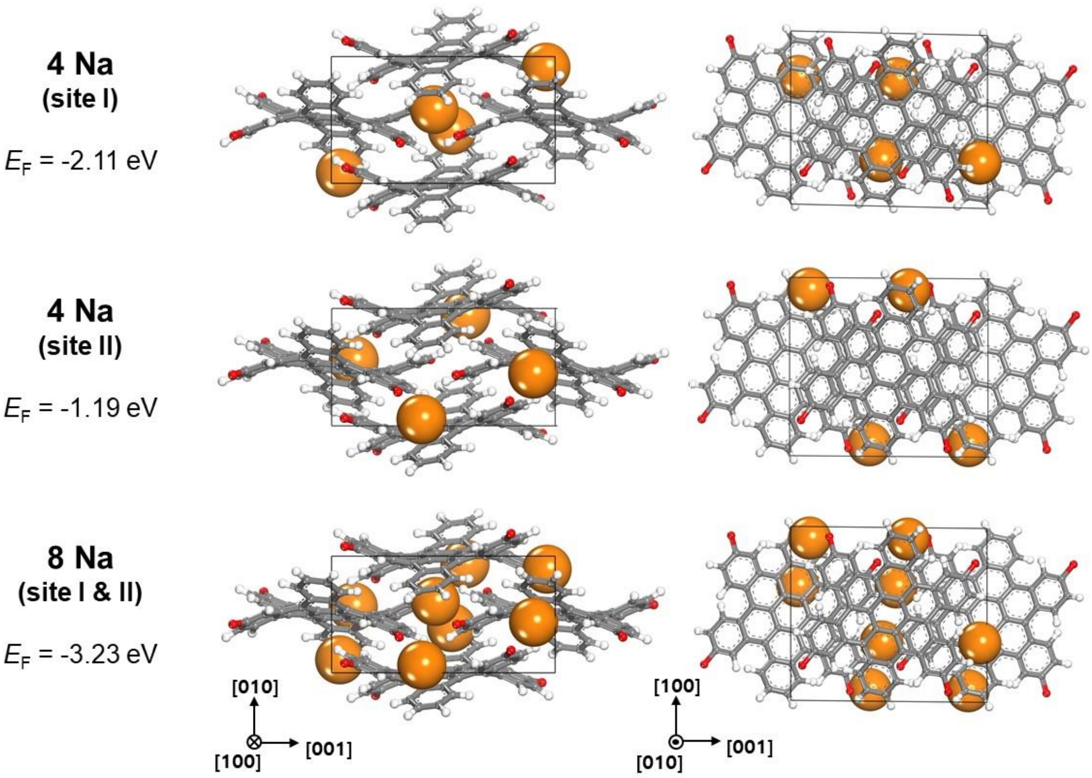

Figure S16. Projection views along the [100] and [010] directions of the F–cHBC structures with 4 and 8 Na-ions adsorbed and their corresponding formation energies. Carbon, hydrogen, fluorine and sodium are colored grey, white, red and orange, respectively.

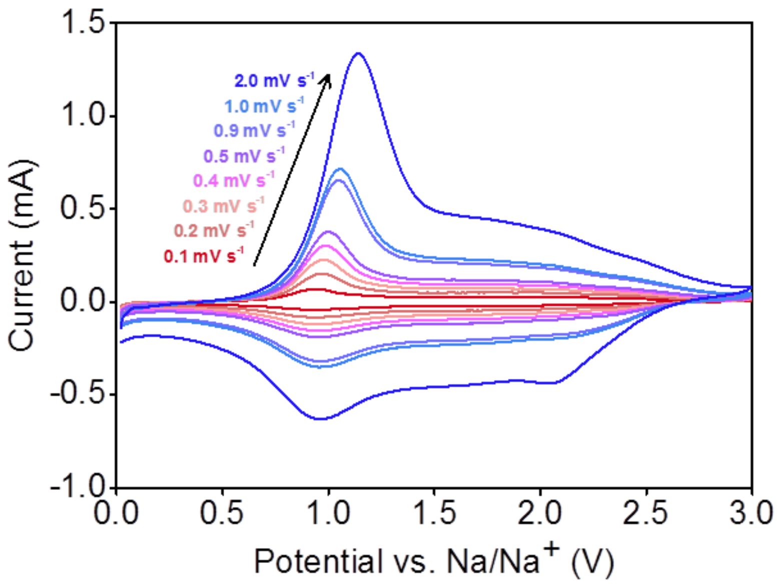

Figure S17. CV of Na-ion stroage cell at various scan rate from 0.1 mV s^{-1} to 2.0 mV s^{-1}.

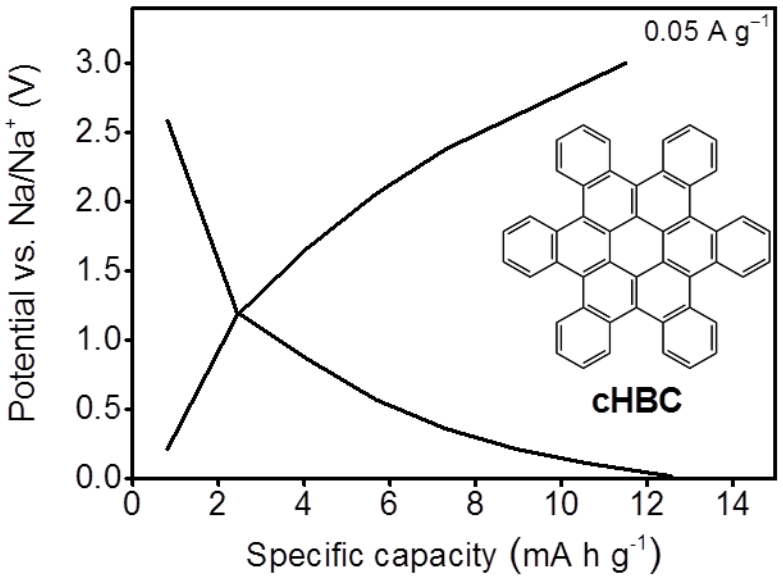

Figure S18. Galvanostatic discharge–charge profiles of THF–330 °C-annealed pristine cHBC anode for Na-ion storage cell at fixed current density of 0.05 A g^{-1}.

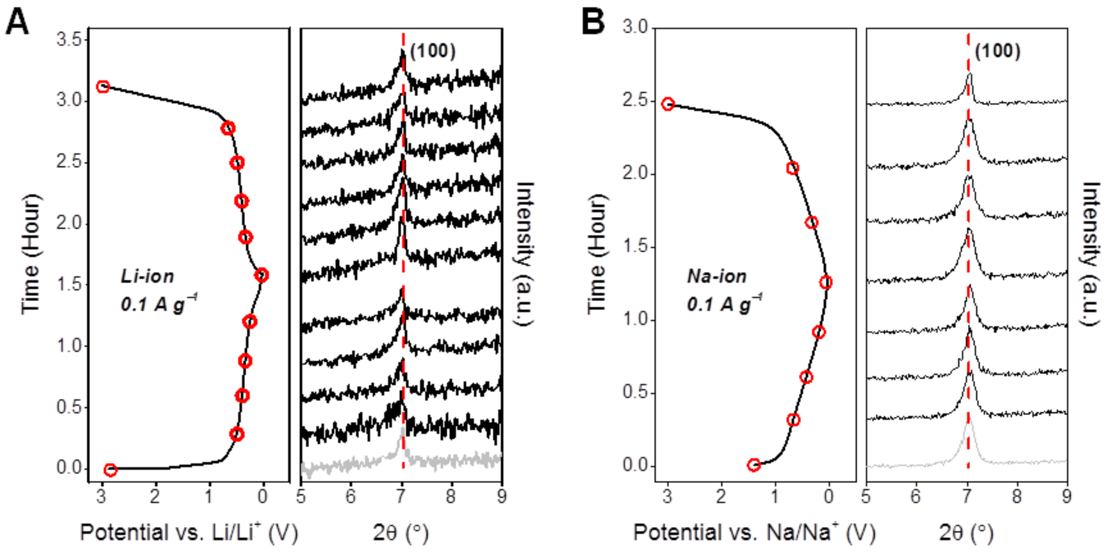

Figure S19. XRD patterns of pristine (Gray line) and galvanostatic discharged-charged A) Li-ion and B) Na-ion cells containing F–cHBC anode.

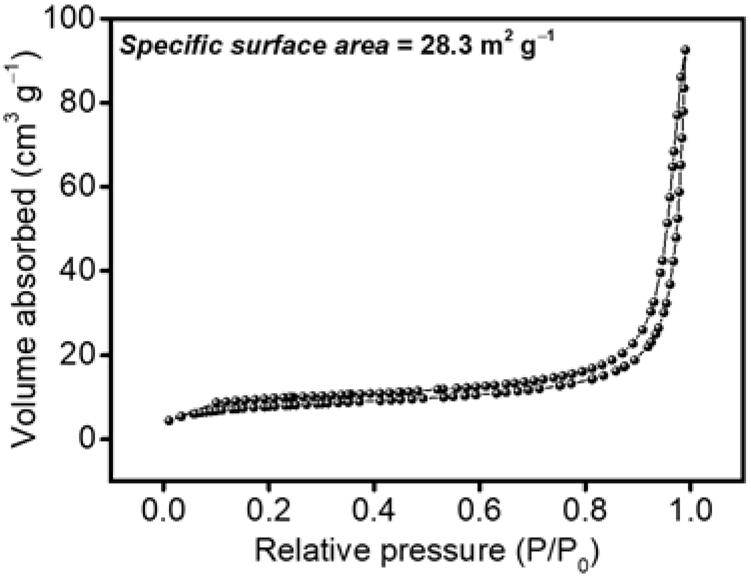

Figure S20. Nitrogen adsorption–desorption isotherm of F–cHBC after 330 °C thermal annealing.
